# Investigation of multiple mortality events in eastern box turtles (*Terrapene carolina carolina*)

**DOI:** 10.1371/journal.pone.0195617

**Published:** 2018-04-05

**Authors:** Laura Adamovicz, Matthew C. Allender, Grace Archer, Marta Rzadkowska, Kayla Boers, Chris Phillips, Elizabeth Driskell, Michael J. Kinsel, Caroline Chu

**Affiliations:** 1 Wildlife Epidemiology Laboratory, University of Illinois College of Veterinary Medicine, Urbana, Illinois, United States of America; 2 Prairie Research Institute, Illinois Natural History Survey, Champaign, Illinois, United States of America; 3 Department of Pathobiology, University of Illinois College of Veterinary Medicine, Urbana, Illinois, United States of America; 4 Zoological Pathology Program, University of Illinois College of Veterinary Medicine, Urbana, Illinois, United States of America; US Geological Survey, UNITED STATES

## Abstract

Wildlife mortality investigations are important for conservation, food safety, and public health; but they are infrequently reported for cryptic chelonian species. Eastern box turtles (*Terrapene carolina carolina*) are declining due to anthropogenic factors and disease, and while mortality investigations have been reported for captive and translocated individuals, few descriptions exist for free-living populations. We report the results of four natural mortality event investigations conducted during routine health surveillance of three Illinois box turtle populations in 2011, 2013, 2014, and 2015. In April 2011, over 50 box turtles were found dead and a polymicrobial necrotizing bacterial infection was diagnosed in five survivors using histopathology and aerobic/anaerobic culture. This represents the first reported occurrence of necrotizing bacterial infection in box turtles. In August 2013, paired histopathology and qPCR ranavirus detection in nine turtles was significantly associated with occupation of moist microhabitats, identification of oral plaques and nasal discharge on physical exam, and increases in the heterophil count and heterophil to lymphocyte ratio (p < 0.05). In July 2014 and 2015, ranavirus outbreaks reoccurred within a 0.2km radius of highly-disturbed habitat containing ephemeral ponds used by amphibians for breeding. qPCR ranavirus detection in five individuals each year was significantly associated with use of moist microhabitats (p < 0.05). Detection of single and co-pathogens (Terrapene herpesvirus 1, adenovirus, and *Mycoplasma* sp.) was common before, during, and after mortality events, but improved sample size would be necessary to determine the impacts of these pathogens on the occurrence and outcome of mortality events. This study provides novel information about the causes and predictors of natural box turtle mortality events. Continued investigation of health, disease, and death in free-living box turtles will improve baseline knowledge of morbidity and mortality, identify threats to survival, and promote the formation of effective conservation strategies.

## Introduction

Wildlife diseases have caused mass mortality events in all vertebrate taxa, including mammals (white-nose syndrome), birds (West Nile virus), fish (infectious salmon anemia), amphibians (chytridiomycosis), and reptiles (snake fungal disease) [1–6]. These mortality events can threaten species survival, and in some cases, lead to extinction [7]. Furthermore, emerging zoonotic diseases have been uncovered during wildlife mortality events (i.e. West Nile virus), highlighting the importance of these outbreaks from a public health perspective [8–9]. Investigation of wildlife disease outbreaks can identify etiologic agents, determine risk factors and population impacts, and lead to management recommendations to avoid or mediate future episodes–potentially impacting wildlife conservation, food security, and human health [8,10–11].

The dynamics of health, disease, and death in wildlife populations are inherently complex, and mortality investigations are most useful when conducted in conjunction with routine health surveillance programs which document “expected” causes and rates of morbidity and mortality [12–15]. Historical data incorporating spatiotemporal factors can help identify patterns of disease and mortality associated with specific demographic groups, stocking densities, and landscape features, all of which can suggest mechanisms for pathogen transmission and maintenance [12,14–15]. Longitudinal data provide a context for identifying mortality events which are larger than expected, attributed to an unusual etiology, or which impact population size and viability. Health datasets which incorporate baseline health surveillance, spatiotemporal factors, and specific mortality investigations from longitudinally sampled populations therefore promote a greater understanding of the proximate causes and drivers of mortality events affecting species survival. Combined mortality investigation and health surveillance programs have been used to identify and address disease-related threats to several endangered species in the United States, including bubonic plague in black-footed ferrets (*Mustela nigripes*), avian vacuolar myelinopathy in bald eagles (*Haliaeetus leucocephalus*), and parasitism in Laysan ducks (*Anas laysanensis*) [16].

While mortality investigations yield valuable information for conservation, they are subject to some important biases. Identification of mortality events is most likely in areas which are highly populated or traveled, and in large, charismatic, or highly visible species [10]. Failure to detect mortality events is more common in remote areas, cryptic animals, and species in which remains are quickly scavenged [10–11,13]. Chelonians represent one of the most imperiled vertebrate groups, with over 60% of species considered threatened or worse [17]. Turtle population stability is highly impacted by removal of mature adults, especially females, due to a combination of delayed sexual maturity and low juvenile survivorship [18]. Mortality events involving multiple individuals may therefore represent an important threat to chelonian population viability. However, due to the small size and cryptic nature of turtles in the family Emydidae, mortality events are infrequently reported and the opportunity to formulate targeted conservation strategies is lost [19].

Eastern box turtles (*Terrapene carolina carolina*) are experiencing range-wide declines due to a combination of anthropogenic factors and disease [20]. Disease-related mortality events, primarily associated with frog virus 3-like ranavirus (FV3), are reported for captive [21–25] and translocated [26–27] box turtles, but few reports involve free-living populations. While FV3 represents a known cause of box turtle mortality, several novel pathogens including Terrapene herpesvirus 1 (TerHV1), box turtle adenovirus (BTADV), and an un-named *Mycoplasma* sp. (BTMyco) have been recently identified [28–33]. The impact of these pathogens on free-living populations and their relationship to mortality events is unclear. Characterization of natural mortality events is an important step towards improving baseline knowledge of box turtle health and disease, identifying potential threats, and forming effective conservation strategies for this species.

We have performed mortality investigations, health assessments, and multi-pathogen surveillance in several populations of Eastern box turtles since 2011. The objectives of this manuscript are to describe four natural mortality events in *T*. *carolina carolina*, to characterize the causes of death in each outbreak, and to identify predictors of mortality.

## Materials and methods

### Ethics statement

Our study involved the use of wild box turtles living on public lands. Permission to work at each public field site was granted by the Illinois Department of Natural Resources, and all animal procedures were approved by the Institutional Animal Care and Use Committee at the University of Illinois (Protocols: 10051 and 13061). Euthanasia of some wild turtles encountered during this study was deemed necessary by a veterinarian (author MC Allender) due to the presence of severe clinical signs and overall grave prognosis for recovery. Euthanasia was carried out by a veterinarian (author MC Allender) using the following American Veterinary Medical Association and IACUC approved protocol: turtles were first heavily sedated using ketamine (100mg/kg IM, MWI Animal Health, Boise, ID 83705) and then euthanized with an overdose of pentobarbital (390mg IV, Vortech Pharmaceutical Ltd, Dearborn, MI 48126).

### Study sites

Box turtle populations were surveyed at Forest Glen Nature Preserve (FGNP [40.01°, -87.57°]), Kennekuk Cove County Park (KCCP [40.19°, -87.72]), and Kickapoo State Park (KSP [40.14°, -87.74]) in east-central Illinois from April-October beginning in 2011. FGNP is an 1800 acre park composed primarily of beech-maple and oak-hickory forest bounded by the Vermillion River. KCCP contains 3000 acres of oak-hickory forest interspersed with small prairies bounded by the Middle Fork National Scenic River. KSP is a 2800 acre park situated on a reclaimed surface mine. It includes relatively young successional forests and the Middle Fork National Scenic River. Invasive plant species are actively removed at FGNP, but they are not removed and have become pervasive at KCCP and KSP. Natural and man-made wetlands are present at all sites.

### Animal populations and sample collection

#### Non-outbreak sampling

Routine turtle health assessment occurred using canine searches one to three times each active season (May, June/July, and September) as previously described [34]. Briefly, teams of 3–4 dogs were used to locate eastern box turtles during a 2–4 hour search period. The search path was equivalent each time a site was visited, but due to the differences in topography, size, and hydrology, sampling efforts were not directly comparable between sites. Capture locations were recorded using global positioning software (GPS) via handheld devices (Garmin International Inc., Olathe, KS, USA). Date, time, and categorical habitat (field, forest, edge) and microhabitat (leaves, grass, brambles, soil, road, moist area) data were also recorded at each turtle location. Microhabitat classification was assigned based on the primary ground cover beneath and within a one foot radius of the turtle. Moist areas consisted of ephemeral bodies of water. Air and substrate temperature were collected at the start and stop of each turtle search, and the results were averaged and recorded for each animal encountered (Kestrel 3000 Weather Meter, Nielsen-Kellerman, Boothwyn, PA 19061; Taylor 9878 Digital Pocket Thermometer, Taylor Precision Products, Oak Brook, IL 60523).

Turtles were weighed to the nearest gram, categorized by sex and age class, and measured (straight carapace length (SCL), carapace height (CH), and straight carapace width (SCW)) to the nearest millimeter. Age class (adult vs. juvenile) was assigned based on carapace length and annuli count. Turtles with a carapace length less than 9 cm (3.5 in) and an annuli count less than or equal to seven were characterized as juveniles [20]. Sex was classified as male, female, or unknown based on plastron concavity and tail length. Blood samples (less than 0.8% body weight) were collected from the subcarapacial sinus into lithium heparin coated microtainers (Becton Dickinson Co., Franklin Lakes, NJ 07417) for hematology and pathogen detection. Swabs of the oral cavity and choana were collected using cotton-tipped plastic handled applicators (Fisher Scientific, Pittsburgh, PA 15275) and stored at -20°C. A single veterinarian evaluated clinical signs (MCA). Physical examination abnormalities were recorded as present (1) or absent (0) for the carapace, plastron, appendages, tympanic membranes, integument, nares, and cloaca. The presence of diarrhea, ectoparasites, and clinical signs of upper respiratory disease (ocular discharge, blepharoedema, nasal discharge, oral discharge, oral plaques, and open mouth breathing) were coded as present (1) or absent (0). The marginal scutes were notched with a unique pattern for permanent identification [35]. Each turtle was released at its original capture site within six hours of initial contact.

#### Outbreak sampling

Outbreaks were identified by park or lab staff and targeted human searches were conducted every 1–3 days by lab staff. Searches were discontinued when 3 consecutive visits to the site failed to identify any new cases. Sampling and evaluation of live turtles was performed as described for non-outbreak turtles. In injured turtles, wound beds were flushed with sterile saline. Aerobic and anaerobic cultures were collected with sterile cotton-tipped applicators, placed into transport media (Remel Stuart Transport Medium, Remel A.C.T. II, Thermo Fisher Scientific, Waltham, MA, USA), and stored at 4°C for 2–4 hours until submission to the University of Illinois College of Veterinary Medicine Veterinary Diagnostic Laboratory. Turtles with severe clinical signs of illness (stuporous mentation, respiratory distress) were hospitalized for supportive care and monitoring. Turtles in moribund condition, or those that declined during hospitalization, were euthanized as described above. Euthanized turtles were either submitted for full necropsy with histopathology, or frozen at -20°C for future testing.

Shells or freshly dead turtles found in the field were collected, double bagged, and transported to the lab on wet ice. GPS locations of the remains and categorical habitat and microhabitat data were recorded as previously described, and each animal was given a unique identification number. Samples of remaining soft tissue were collected, if present, and banked at -80°C for future testing. Shells were stored at -20°C. In 2015, a bone marrow sample was collected from the right bridge of each shell using a hand-held drill with sterilized bits [3]. Bone marrow samples were stored at -20°C until DNA extraction.

Live and dead amphibians were opportunistically collected from moist areas at each study site during visual encounter surveys as part of an ongoing demographic study. Up to five individuals of each species were humanely euthanized using an overdose of buffered tricaine methylsufate (3-Aminobenzoic Acid Ethyl Ester, Sigma Chemical Co. St. Louis, MO 61378) for vouchering with the Illinois Natural History Survey and either frozen at -20°C or preserved in 100% ethanol (Decon Laboratories Inc., King of Prussia, PA 19406). In 2013, samples of liver and kidney were harvested from amphibians collected within the same stream as five deceased box turtles and stored at -20°C for future qPCR pathogen testing.

### Sample analysis

#### Hematology

Packed cell volume (PCV) was determined using sodium heparinized microhematocrit tubes (Jorgensen Laboratories,Inc., Loveland, CO 80538) centrifuged at 14,500 rpm for five minutes. Total solids was determined with a hand-held refractometer (Amscope RHC-200ATC refractometer, National Industry Supply, Torrance, CA, USA) using plasma from the microhematocrit tube. Total white blood cell (WBC) counts were determined using an Avian Leukopet (Vet lab Supply, Palmetto Bay, FL, USA) on a Bright-line hemacytometer (Hausser Scientific, Horsham, PA, USA) following the manufacturer’s protocol. Blood smears made from lithium heparinized microtainers were stained with a modified Wright’s Giemsa stain (Diff-quik) and used for differential leukocyte counts.

#### Microbiology

Aerobic culture swabs were plated on Columbia blood agar, Colistin, Naladixic Blood Agar (CNA), and MaConkey Agar (Remel, Thermo Fisher Scientific, Waltham, MA, USA) both at 37°C with 5% CO_2_, and at 25°C in room air. Anaerobic culture swabs were plated on Brucella Agar, Laked blood w/ kanamycin, vancomycin (LKV Agar), and phenol ethyl alcohol agar (PEA) (Remel, Thermo Fisher Scientific, Waltham, MA, USA) at 37°C in anaerobic gas mixture. Organisms were identified using gram negative identification plate (GNID) and/or gram positive identification plate (GPID) panels (VersaTREK and Sensititre, Thermo Fisher Scientific, Waltham, MA, USA). Fastidious and/or non-fermenting gram negative organisms were identified using GN2 microplate panels (Biolog, Hayward CA, USA). Anaerobes were identified by the Wadsworth Disk method [36] or anaerobic identification test panel microplates (Biolog, Hayward CA, USA).

#### Virus isolation

Frozen whole blood (1mL) was added to 5mL erythrocyte lysis buffer (Buffer EL, Qiagen, Valencia, CA, USA), vortexed for 15 seconds, and incubated on ice for 30 minutes with additional vortexing every 15 minutes. The lysed sample was centrifuged at 1700rpm for 10 minutes at 4°C and the supernatant was discarded. The pellet was resuspended in 2mL Minimum Essential Medium (MEM, Thermo Fisher Scientific, Waltham, MA, USA) with 5μg/mL amphotericin B, 200U/mL penicillin, 200μg/mL streptomycin, and 100μg/mL gentamycin (Sigma Chemical Co. St. Louis, MO 61378). This was inoculated onto Terrapene heart cells (TH-1) grown to 80% confluence in 75 cm^3^ flasks. Following a 10 minute incubation at 27°C, 20mL of Dulbecco’s modified eagle medium (DMEM, Thermo Fisher Scientific, Waltham, MA, USA) with 10% fetal bovine serum (FBS, Thermo Fisher Scientific, Waltham, MA, USA), 100U/mL penicillin, 100μg/mL streptomycin, and 2.5μg/mL amphotericin B was added to each flask, and cells were maintained at 27°C with 5% CO_2_. If necessary, blind passaging was performed after inoculated cells reached 100% confluency. Flasks were frozen at -80°C, thawed, and vortexed three times. One milliliter of the flask contents was used to inoculate a new flask containing TH-1 grown to 80% confluency. Infected flasks were monitored daily for signs of cytopathic effects (CPE).

#### Molecular diagnostics

DNA was isolated from oral swabs, whole blood, virus isolation flasks, bone marrow, and amphibian tissue samples using a commercially available kit (QIAmp DNA Blood Mini Kit and DNAeasy kit, Qiagen, Valencia, CA, USA). Manufacturer instructions were followed with some alterations for bone marrow samples, as previously described [37]. DNA concentration and purity was assessed spectrophotometrically (NanoDrop 1000, Thermo Fisher Scientific, Waltham, MA, USA) and DNA samples were stored at -80°C. Turtle oral swab DNA samples were assayed for FV3, BTADV, BTMyco, and TerHV1. Turtle whole blood, bone marrow, and amphibian tissues were tested for FV3. Testing for FV3, TerHV1, and BTADV was performed using existing TaqMan qPCR assays [38–40]. Briefly, reactions were run in triplicate using a real-time PCR thermocycler (7500 ABI realtime PCR System, Applied Biosystems, Carlsbad, CA). Real-time qPCR data analysis and quantification was performed using commercially available software (Sequence Detection Software v2.05, Applied Biosystems, Carlsbad, CA). Quantification was performed by comparing each sample’s cycle threshold (Ct) value to a standard curve consisting of purified, PCR generated target segments of each pathogen’s genome (seven tenfold dilutions from 10^8^–10^1^ copy numbers).

A novel conventional PCR assay targeting the 16S rRNA gene was developed and used to screen for BTMyco using the forward 5’ GGAGAATCTCGCTAACGCAG 3’ and reverse 5’ AGCCTTCAATCCGAACTGAG 3’ primer set. Each 50μL reaction included 36.5μL sterile deionized water, 5μL 10x buffer, 4μL 50mM magnesium chloride, 1μL 10mM dNTPs, 0.5μL Taq polymerase (5U/μL), and 1μL of each 10μM primer (Taq DNA Polymerase 500U, Invitrogen, Carlsbad, CA 92008). Thermocycler settings consisted of a five minute initial denaturation at 95°C followed by 35 cycles of 95°C for 45 seconds, 59°C for 60 seconds, and 72°C for 120 seconds, then a final elongation step of 72°C for seven minutes.

A 470 base pair product obtained from an eastern box turtle oral swab was sequenced in both directions (W.M. Keck Center for Comparative and Functional Genomics, University of Illinois at Urbana-Champaign, Urbana, IL) and compared to known sequences in GenBank using BLASTN to confirm consistency with BTMyco. This PCR product was cloned into *Escherichia coli* (TOPO TA Cloning Kit, Invitrogen, Carlsbad, CA), and plasmids were purified using a commercially available kit (QIAfilter plasmid Maxi Kit, Qiagen, Valencia, CA). Cloning products were verified via sequencing and plasmids were linearized using EcoRI (Clontech Laboratories, Mountain View, CA). Linearized plasmids were phenol-chloroform precipitated and DNA concentration and purity was assessed spectrophotometrically. Bacterial copy number was calculated using the following formula:

$$\#copies/µl=\frac{\left(plasmid+insert\;ng/µl)*(6.022\;x\;10^{23}\;copies/mol\right)}{\left(base\;pair\;length)(1\;x\;10^{9}\;ng/g)(650\;g/mol\;of\;base\;pair\right)}$$

Assay sensitivity and limit of detection was evaluated using the BTMyco plasmid serially diluted from 10^9^−10^0^ copies. Specificity was assessed using sequence-confirmed positive samples for *M*. *agassizii*, *M*. *testudineum*, *M*. *canis*, *M*. *hyorrhinus*, *M*. *hyopneumoniae*, *M*. *bovis*. Oral swab DNA collected during routine health assessments and outbreaks was assayed. PCR products of the appropriate size were sequenced and blasted to confirm accurate detection. All *Mycoplasma* sp. assays included a positive control (plasmid) and a negative control (water).

Conventional PCR assays targeting the major capsid protein (MCP) gene were utilized to confirm FV3 growth in virus isolation, as previously described [41]. Each assay included a sequence-confirmed FV3 positive sample as a positive control, and water as a negative control. Bands of the appropriate size (approximately 500bp) were sequenced in both directions and compared to existing sequences in Genbank using BLASTN.

### Mapping and spatio-temporal analyses

GPS locations of turtles and amphibians were mapped using ArcGIS on the World Imagery background layer (ArcMap 10.4.1, Esri, Redlands, CA 92373). Hydrology data were obtained from the Illinois Geospatial Data Clearinghouse (https://clearinghouse.isgs.illinois.edu/, accessed March 2017) and a 2015 Illinois highway shapefile was obtained from the Illinois Department of Transportation (http://www.idot.illinois.gov/transportation-system/local-transportation-partners/gis-data-share, accessed 3/10/17). Both were uploaded into ArcMap, and the “Near” tool was used to calculate the distance from each turtle’s capture site to the nearest road, and permanent body of water. The Bernoulli model was used to test for statistically significant purely spatial or spatio-temporal clusters of disease using SaTScan v9.4.4 [42]. Output files from SaTScan were uploaded into ArcGIS and overlayed onto turtle and amphibian maps to visualize the extent of significant clusters.

### Statistical analysis

All turtles evaluated within the same study site and year were assigned to the following three categories: pre-outbreak (PreOB), outbreak (OB), and post-outbreak (PostOB). An outbreak was defined as the time period encompassing the first and last death due to a common etiology. Turtles evaluated before the first death were classified as PreOB, while turtles assessed after the last death were assigned to the PostOB group. The effects of outbreak grouping on pathogen detection status and hematology parameters were evaluated using logistic and linear regression, respectively. The mean, standard deviation, median, range, and distribution (normal vs. non-normal as assessed using skewness, kurtosis, and the Shapiro-Wilk statistic) were determined for each hematology parameter and tabulated based on outbreak grouping.

Turtles within the same site and year were then re-grouped based on detection of the pathogen responsible for the mortality event–i.e. the cause of death (considered categorically–case or non-case). Logistic regression was used to evaluate the effects of categorical (habitat, microhabitat, sex, age class) and continuous predictors (hematology parameters, weight, distance metrics) on detection of the cause of death. Odds ratios were determined by exponentiating the coefficient estimates of statistically significant logistic regression models. An alpha value of 0.05 was used to establish statistical significance. All statistical evaluations were performed in R v 3.2.3 [43].

## Results

### Population demographics, habitat use, physical exam, and timeline

**Fig 1:**
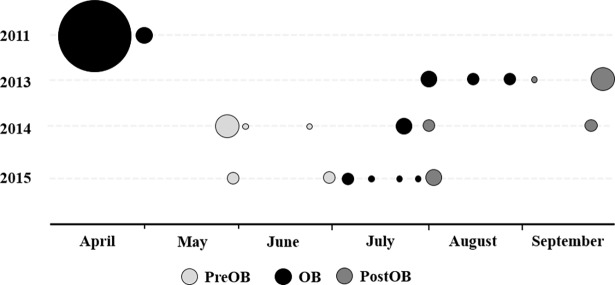
Number and timing of Eastern box turtle (*Terrapene carolina carolina*) captures before (PreOB), during (OB), and after (PostOB) mortality events in Vermilion County, Illinois from 2011–2015. The size of the marker reflects the number of animals captured.

**Fig 2:**
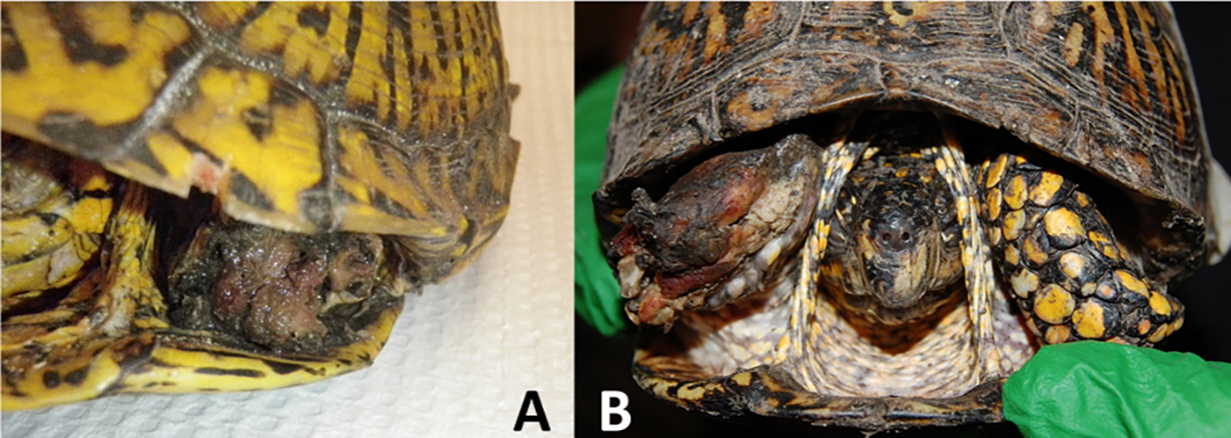
Gross appearance of necrotizing bacterial infection in *Terrapene carolina carolina* from Forest Glen Nature Preserve, Vermilion County, Illinois during a mortality event in 2011. Left forelimb (A) and right forelimb (B).

In April 2011, fifty-three box turtle shells were encountered by FGNP park personnel. Subsequently, we conducted three searches from April 26th–May 2nd, and 12 live turtles were evaluated. Demographics, habitat use, physical examination findings, and timeline are summarized in Table 1 and Fig 1. Five of the live turtles were missing one or more appendages with necrosis of the proximal limb tissues (Fig 2). Two animals were humanely euthanized and submitted for necropsy. Aerobic and anaerobic cultures were collected from the wound beds of four affected turtles, including the necropsied animals.

**Table 1:** Demographics, habitat use, and physical examination abnormalities in Eastern box turtles (*Terrapene carolina carolina*) captured before (PreOB), during (OB), and after (PostOB) mortality events in Vermilion County, IL from 2011–2015. NR = not recorded.

Year	Group	# Dead	# Live	Adult Female	Adult Male	Adult Unknown	Juvenile	Microhabitat	Clinical Signs
**2011**	OB	53	12	5	7	49	4	NR	Limb Necrosis (N = 5)
**2013**	OB	6	9	3	0	9	3		Depression (N = 6)
									Oral Plaques (N = 6)
								Moist Area (N = 14)	Nasal Discharge (N = 3)
								Brambles (N = 1)	Blepharoedema (N = 2)
									Ocular Discharge (N = 2)
									Diarrhea (N = 2)
	PostOB	5	14	2	6	8	3	Leaves (N = 7)	Mild Scute Trauma (N = 3)
								Moist Area (N = 5)	Missing Tail/Foot (N = 2)
								Brambles (N = 1)	Asymmetrical Nares (N = 1)
								NR (N = 4)	Oral Plaques (N = 1)
**2014**	PreOB	1	21	12	7	3	0	Grass (N = 16)	Mild Scute Trauma (N = 5)
								Moist Area (N = 4)	
								Leaves (N = 2)	
	OB	4	6	2	1	6	1	Moist Area (N = 8)	Depression (N = 4)
								Grass (N = 2)	
	PostOB	0	5	3	2	0	0	Leaves (N = 3)	Asymmetrical Nares (N = 1)
								Grass (N = 2)	
**2015**	PreOB	1	4	1	3	1	0		Plastron Erosions (N = 2)
								Leaves (N = 3)	Diarrhea (N = 1)
								Grass (N = 2)	Asymmetrical Nares (N = 1)
									Missing Foot/Digits (N = 2)
	OB	5	0	0	0	5	0	Moist Area (N = 3)	NA
								Grass (N = 1)	
								Leaves (N = 1)	
	PostOB	0	13	4	8	1	0	Brambles (N = 5)	Mild Scute Trauma (N = 2)
								Leaves (N = 5)	Missing Digits (N = 1)
								Grass (N = 3)	Missing Tail (N = 1)

**Fig 3:**
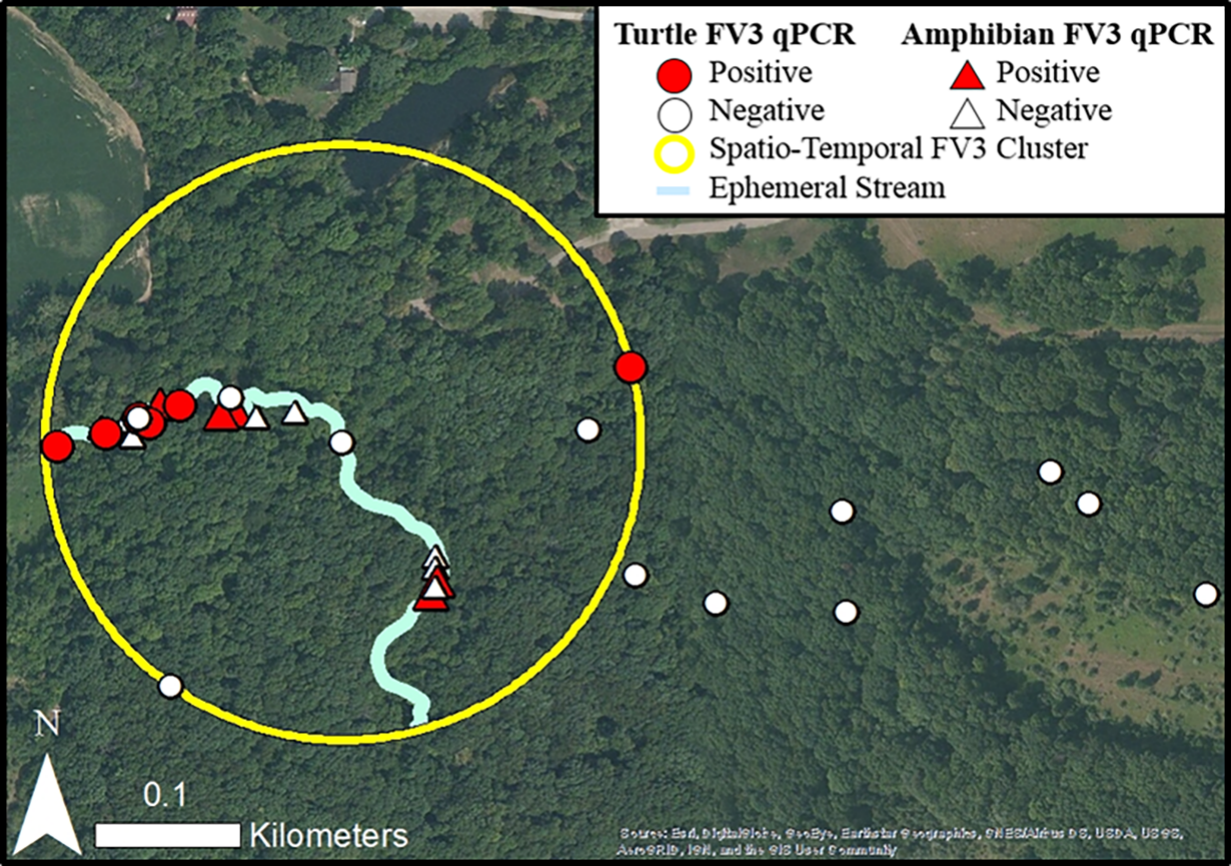
Locations of sympatric Eastern box turtles (*Terrapene carolina carolina*) and amphibians (*Acris crepitans*, *Rana clamitans*, *Eurycea bislineata*) at Kennekuk Cove County Park in Vermilion County, Illinois during and after a natural ranavirus (FV3) outbreak in 2013. A 0.16km spatio-temporal cluster of FV3 cases was identified from August 2^nd^–August 16^th^ in association with an ephemeral stream.

In late July 2013, six dead box turtles were identified by park personnel within an ephemeral stream at KCCP. Subsequently, we conducted five searches from August 2nd–August 29th and nine turtles were encountered (Table 1; Figs 1 and 3). On August 16th, five apparently healthy cricket frogs (*Acris crepitans*), three green frogs (*Rana clamitans*), and five two-lined salamanders (*Eurycea bislineata*) from the same ephemeral stream were euthanized and liver and kidney samples were collected for FV3 testing. The PostOB group included one turtle opportunistically sampled on September 5th and 18 turtles (13 live, five shells) evaluated during routine health surveillance on September 30th.

**Fig 4:**
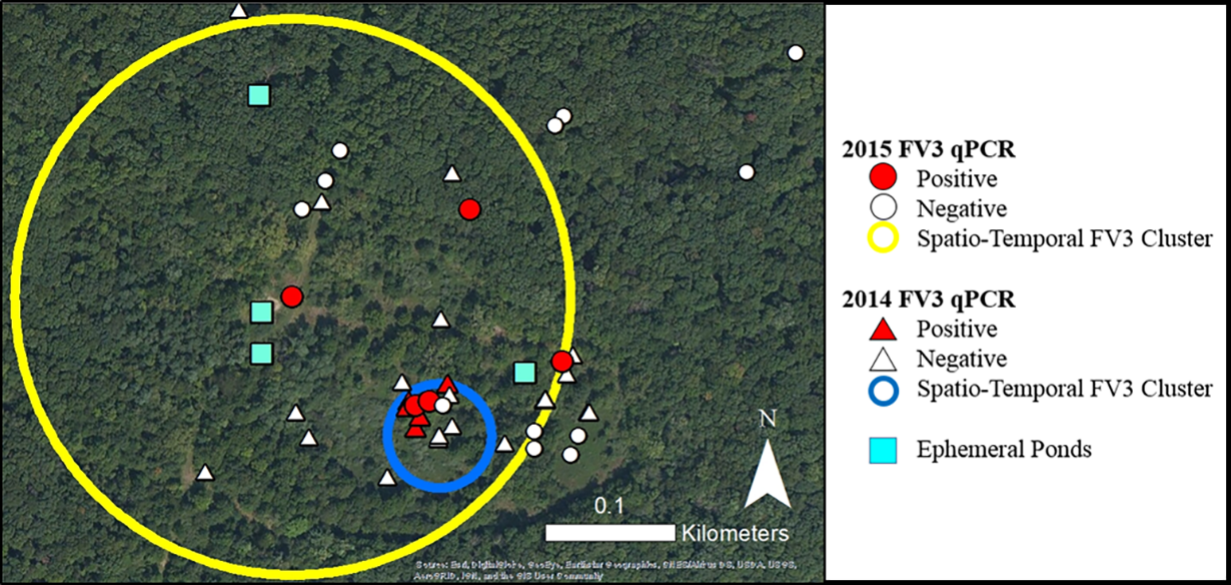
Locations of Eastern box turtles (*Terrapene carolina carolina*) and ephemeral ponds used for amphibian breeding at Kickapoo State Park in Vermilion County, Illinois in 2014 and 2015 before, during, and after ranavirus (FV3) outbreaks. In 2014 a 0.03km spatio-temporal cluster of FV3 cases was identified from July 15^th^–July 23^rd^. In 2015 a 0.16km spatio-temporal cluster of FV3 cases was identified from July 8^th^–July 23^th^.

From July 16-17th 2014, four deceased turtles were discovered at KSP. Six turtles were located during daily searches from July 16-25^th^ (Table 1, Figs 1 & 4). Four depressed individuals were hospitalized for supportive care and monitoring consisting of a thermal gradient and daily warm water soaks. Two of the hospitalized turtles died and two were euthanized after 15–57 days due to progression of clinical signs and moribund condition. Pre-mortality health surveillance was conducted for 20 live box turtles on May 21^st^, one turtle opportunistically sampled on May 30^th^ and a shell found on June 19^th^. Post-outbreak surveillance was conducted from July 29^th^–September 17^th^ and five turtles were evaluated (Table 1).

On July 8th 2015, two freshly deceased box turtles were found during routine health surveillance at KSP within 0.2km of the 2014 mortality event. Mortality investigation was conducted from July 8-28^th^. Three additional shells were recovered, but no living turtles were located (Figs 1 & 4). Pre-mortality investigation was conducted on May 21^st^ and June 30^th^. Four live adult turtles and one freshly dead shell were evaluated (Table 1). Post-mortality investigation was conducted at the end of July and 13 adult turtles were sampled (Table 1).

### Pathogen detection, pathology, and cause of death

The novel BTMyco conventional PCR successfully amplified 10^1^−10^9^ bacterial copies, resulting in a lower limit of detection of 10 copies. The assay produced appropriately-sized bands for BTMyco, *M*. *agassizii*, and *M*. *testudineum*. Bands larger and smaller than the 470bp target were produced for all other *Mycoplasma* species tested. Sequencing of products producing appropriately-sized bands is necessary to distinguish between BTMyco, *M*. *agassizii*, and *M*. *testudineum*. All BTMyco-positive box turtle sequences were 99–100% identical to *Mycoplasma* sp. sequences in Genbank (accession numbers FJ159564, KJ623622-KJ623625).

During the 2011 outbreak at FGNP, BTMyco was detected in two of the 12 live turtles, one with carapacial trauma and one with no clinical signs (Table 2). BTADV was detected in one turtle that was euthanized and necropsied, but intranuclear inclusion bodies were not identified histologically [32]. Gross necropsy findings for both submitted turtles included missing limbs with discoloration and necrosis of the remaining soft tissues. Regionally extensive subacute necrotizing heterophilic osteomyelitis, myositis, cellulitis, and dermatitis with intralesional mixed bacteria, hemorrhage and edema were identified on histopathology. Intravascular, extracellular bacteria (sepsis) was also present. Primary necrotizing bacterial infection was considered the most likely cause of the lesions. The presence of a morphologically and microbiologically (alpha *Streptococcus* sp., *Aeromonas hydrophila*, *Corynebacterium* sp., and *Bacteroides* sp.) similar bacterial population was suggestive of a common etiology.

**Table 2:** Number of turtles with pathogens detected and cause of death in free living *Terrapene carolina carolina* encountered before (PreOB), during (OB), and after (PostOB) mortality events in east-central Illinois from 2011–2015. Turtles with single pathogen detections are counted in the individual pathogen columns. Turtles with co-pathogens are counted in the co-infections column. FV3 = Frog virus 3-like ranavirus, BTADV = box turtle adenovirus, TerHV1 = Terrapene herpesvirus 1, BTMyco = *Mycoplasma* sp., NBI = necrotizing bacterial infection.

Year	Group	# Tested	FV3	TerHV1	BTMyco	BTADV	Co-Infections	Cause of Death
**2011**	OB	61	0	0	2	1	0	NBI
**2013**	OB	13	6^a^	0	2	0	2 (FV3 + BTMyco)	FV3
							1 (FV3+BTMyco+TerHV1)	
	PostOB	16	0	1	0	0	1 (TerHV1 + BTADV)	
							1 (TerHV1 + BTMyco)	
**2014**	PreOB	13	0	2	1	0	3 (TerHV1 + BTMyco)	FV3
							1 (TerHV1+BTADV+BTMyco)	
	OB	8	3^b^	0	0	0	1 (FV3 + TerHV1)	
							1 (FV3 + BTMyco)	
							1(TerHV1 + BTMyco)	
	PostOB	5	0	5	0	0	0	
**2015**	PreOB	5	0	0	1	0	0	FV3
	OB	5	5	0	0	0	0	
	PostOB	13	0	0	0	1	0	

^a^ 4 live turtles, 2 shells

^b^ 1 live turtle, 2 shells

In 2013, all outbreak turtles in which FV3 was detected as a sole pathogen (N = 4) presented with oral plaques (Table 1). One of these animals also had nasal discharge and another had diarrhea, blepharoedema, and depressed mentation. Turtles with FV3 and BTMyco co-detection (N = 2) presented with oculonasal discharge and blepharoedema or oral plaques and inactivity. A single turtle co-infected with FV3, TerHV1, and BTMyco had oral plaques and diarrhea. Turtles in which BTMyco was the sole pathogen detected (N = 2) displayed oculonasal discharge or oral plaques, extreme pallor, and depressed mentation. Five turtles evaluated with necropsy and histopathology exhibited one or more lesions consistent with ranavirus infection including: fibrinoid necrosis in the splenic vessels with or without fibrinoid necrosis in various other organs (3 turtles) and ulcerative stomatitis, esophagitis, and/or gastritis (3 turtles). The cause of the mortality event was determined to be FV3. FV3 was also detected from liver and kidney samples of apparently healthy amphibians, including two sympatric *A*. *crepitans* (40%), one *R*. *clamitans* (33.3%), and two *E*. *bislineata* (40%).

The odds of FV3 and BTMyco detection were 20 (p = 0.008) and 15 (p = 0.02) times higher during the 2013 outbreak compared to PreOB and PosOB, and the odds of testing negative for all pathogens were 33 (p = 0.004) times higher PostOB. The odds of FV3 detection were 18.6 times higher (p = 0.02) in turtles occupying a moist microhabitat, 35 times higher in turtles with oral plaques (p = 0.007), 15 times higher in turtles with nasal discharge (p = 0.03), and 12 times higher in turtles with any physical exam abnormality (p = 0.03). The odds of FV3 detection also rose 1.5 times with every 1 unit increase in the heterophil/lymphocyte ratio (HL), a change primarily driven by a disproportionate increase in relative heterophil count (p = 0.03).

FV3 was detected as a single or co-infection in four live OB turtles and two shells from KSP in 2014 (Table 1). Live FV3 was isolated from the blood of one 2014 turtle. CPE consisting of cellular rounding and detachment was apparent after a single blind passage, and sequencing of the MCP gene was 100% identical to an FV3 sequence in Genbank (accession number KJ175144.1). Hospitalized FV3 positive turtles survived 15–37 days while one individual with TerHV1 and BTMyco co-detection lived for 57 days. Several single and co-pathogens were detected in the PreOB group, but only TerHV1 was detected in the PostOB group (Fig 3, Table 2). The cause of the mortality event was determined to be FV3. The odds of FV3 detection were 22 times higher in the OB group compared to the PreOB group (p = 0.009) and 23.8 times higher in turtles occupying a moist microhabitat (p = 0.007).

At KSP in 2015, FV3 was detected in the bone marrow from all OB turtle shells, and the cause of death was determined to be FV3 (Table 2). The odds of FV3 detection were 25 times (p = 0.03) and 65 times (p = 0.005) higher in the OB group compared to the PreOB and PostOB groups, respectively. The odds of FV3 detection were 45 times higher (p = 0.004) in turtles occupying a moist microhabitat.

### Hematology

In 2011, significant hematological differences were not detected between turtles with necrotizing infections and apparently healthy turtles (Table 3). The adenovirus positive turtle had the highest total leukocyte count and monocyte count of all OB turtles.

**Table 3:** Complete blood counts for free-living *Terrapene carolina carolina* encountered during a mortality event in Forest Glen Nature Preserve, Vermilion County, Illinois in 2011.

Parameter	N	Mean	SD	Median	Minimum	Maximum	Distribution
**PCV (%)**	10	18.85	7.56	19.5	9	34.5	Normal
**TS (g/dL)**	10	2.96	1.46	2.87	1	5.4	Normal
**WBC (x10**^**3**^**/**μ**L)**	10	4.939	2.654	5.028	0.605	9.166	Normal
**Heterophils (x10**^**3**^**/**μ**L)**	10	2.179	1.402	2.137	0.157	4.626	Normal
**Lymphocytes (x10**^**3**^**/**μ**L)**	10	1.152	1.014	0.881	0.109	2.836	Normal
**Monocytes (x10**^**3**^**/**μ**L)**	10	1.049	1.158	0.796	0.136	4.125	Non-Normal
**Eosinophils (x10**^**3**^**/**μ**L)**	10	0.192	0.129	0.212	0.036	0.39	Normal
**Basophils (x10**^**3**^**/**μ**L)**	10	0.22	0.242	0.143	0	0.845	Non-Normal
**Heterophil/Lymphocyte**	10	4.275	6	2.19	0.3	20.5	Non-Normal

CBCs were performed for four outbreak turtles and thirteen post-outbreak turtles in 2013 (Table 4). Heterophils (p < 0.001) and the HL ratio (p = 0.004) were significantly higher in the outbreak turtles compared to the post-outbreak turtles, while the difference in total leukocyte count and monocyte count approached significance (p = 0.08). One FV3 positive OB turtle with oral plaques had the highest values for all leukocytes, while the FV3 positive turtle with diarrhea had the lowest TS. The turtle with FV3, BTMyco, and TerHV1 co-detection had the lowest values for all leukocytes and the highest HL ratio compared to other OB turtles. A single PostOB turtle with TerHV1, asymmetrical nares, and a missing rear foot had the highest HL ratio of any PostOB turtle.

**Table 4:** Complete blood counts for free-living *Terrapene carolina carolina* encountered during and after a frog virus 3-like ranavirus mortality event in Kennekuk Cove County Park, Vermilion County, Illinois in 2013.

				Outbreak						Post-Outbreak				
Parameter	N	Mean	SD	Median	Min	Max	Distribution	N	Mean	SD	Median	Min	Max	Distribution
**PCV (%)**	5	20.2	9.52	23	4	27	Normal	13	19.9	5.3	20	7	27	Normal
**TS (g/dL)**	8	4.05	1.07	4.2	2	5.2	Normal	13	3.66	1.03	3.6	2.2	6.4	Normal
**WBC (x10**^**3**^**/mL)**	4	18.094	14.777	13.807	5.346	39.418	Normal	13	9.755	4.838	7.98	2.337	17.132	Non-Normal
**Heterophils (x10**^**3**^**/mL)**^**a**^	4	9.176	5.844	8.032	3.688	16.95	Normal	13	2.308	1.46	2.107	0.631	6.419	Non-Normal
**Lymphocytes (x10**^**3**^**/mL)**	4	1.405	0.882	1.567	0.267	2.219	Normal	13	1.15	0.821	0.872	0.397	2.845	Non-Normal
**Monocytes (x10**^**3**^**/mL)**	4	2.211	3.522	0.544	0.267	7.489	Non-Normal	13	0.494	0.475	0.335	0	1.422	Non-Normal
**Eosinophils (x10**^**3**^**/mL)**	4	3.711	4.165	2.201	0.588	9.854	Normal	13	4.226	2.657	3.102	0.794	8.663	Normal
**Basophils (x10**^**3**^**/mL)**	4	1.589	1.267	1.335	0.534	3.153	Normal	13	1.575	1.301	1.041	0.187	3.7	Non-Normal
**Heterophil/Lymphocyte**^**a**^	4	8.45	4.56	8.67	2.65	13.8	Normal	13	2.75	2.433	1.93	0.8	10	Non-Normal

^a^ Statistically significant difference between outbreak and post-outbreak turtles

CBC data were available for eleven of the pre-outbreak turtles and four of the post-outbreak turtles in 2014 (Table 5). Heterophils were significantly higher in the PreOB group than the PostOB group (p = 0.03). The PreOB turtle with TerHV1, BTMyco, and BTADV co-detection had the lowest WBC count and lymphocyte count of its group, but had no clinical signs of illness. In 2015, complete CBCs were performed for three PreOB turtles and twelve PostOB turtles (Table 4). Eosinophils (p = 0.001) and WBC (p = 0.01) were significantly lower in the PreOB group compared to the PostOB group. The PreOB turtle with BTMyco detection and diarrhea had the highest values for WBC, basophils, eosinophils, and lymphocytes and the lowest heterophil and HL ratio values of its group. WBC (p = 0.01) and heterophils (p = 0.002) were significantly higher in the 2014 PreOB group compared to the 2015 PreOB group, but no other differences were noted at KSP between years.

**Table 5:** Complete blood counts for free-living *T*. *carolina carolina* encountered before and after recurrent frog virus 3-like ranavirus mortality events in Kickapoo State Park, Vermilion County, Illinois in 2014 and 2015. CT = measure of central tendency (mean or median), Dispersion = measure of dispersion (standard deviation or 10-90^th^ percentiles).

	2014 PreOB		2014 PostOB		2015 PreOB		2015 PostOB	
Parameter	N	CT; Dispersion	N	CT; Dispersion	N	CT; Dispersion	N	CT; Dispersion
		(Range)		(Range)		(Range)		(Range)
**PCV (%)**	12	**24.25; 4.18**^**N**^	4	**25.63; 5.96**^**N**^	3	**22.8; 2.93**^**N**^	13	**22.7; 4.3**^**N**^
		(17.5–30)		(19–33.5)		(19.5–25)		(13.5–30.5)
**TS (g/dL)**	12	**5.34; 1.21**^**N**^	4	**6.85; 1.11**^**N**^	3	**6.1; 1.83**^**N**^	13	**5.49; 1.5**^**N**^
		(3.85–7.75)		(5.65–7.85)		(4.2–7.85)		(3.1–8.9)
**WBC (x10**^**3**^**/**μ**L)**^**b**^^,^ ^**c**^	11	**12.46; 3.92**^**N**^	4	**10.43; 4.32**^**N**^	3	**5.82; 5.8–6.4**^**NN**^	12	**13.13; 4.43**^**N**^
		(6.26–19.8)		(6.29–15.65)		(5.8–6.53)		(7.36–19.06)
**Heterophils (x10**^**3**^μ**L)**^**a**^^,^ ^**c**^	11	**5.35; 1.6**^**N**^	4	**3.25; 1.04**^**N**^	3	**1.56; 1.18**^**N**^	12	**4.11; 2.08**^**N**^
		(2.82–7.65)		(1.72–4.03)		(0.72–2.9)		(1.78–8.07)
**Lymphocytes (x10**^**3**^**/**μ**L)**	11	**1.2; 0.27–1.7**^**NN**^	4	**0.88; 0.37**^**N**^	3	**1.22; 0.66–1.24**^**NN**^	12	**1.4; 0.73–3.15**^**NN**^
		(0.25–4.94)		(0.53–1.4)		(0.52–1.24)		(0.66–7.43)
**Monocytes (x10**^**3**^**/**μ**L)**	11	**0.74; 0.65**^**N**^	4	**0.16; 0.15**^**N**^	3	**0.48; 0.24**^**N**^	12	**0.71; 0.5**^**N**^
		(0–2.18)		(0–0.37)		(0.23–0.7)		(0.09–1.63)
**Eosinophils (x10**^**3**^**/**μ**L)**^**b**^	11	**2.75; 1.52**^**N**^	4	**4.22; 2.62**^**N**^	3	**2.26; 0.25**^**N**^	12	**5.22; 1.25**^**N**^
		(1.13–6.02)		(1.57–6.89)		(1.97–2.41)		(2.65–7.17)
**Basophils (x10**^**3**^**/**μ**L)**	11	**2.26; 1.51**^**N**^	4	**1.91; 2.43**^**N**^	3	**0.76; 0.77**^**N**^	12	**1.12; 0.91**^**N**^
		(0.6–5.15)		(0.25–5.48)		(0.17–1.63)		(0.08–3.28)
**Heterophil/Lymphocyte**	11	**4.85; 2.74–16.9**^**NN**^	4	**4.45; 2.52**^**N**^	3	**2.33; 2.79**^**N**^	12	**2.83; 1.6**^**N**^
		(0.57–16.67)		(1.22–2.44)		(0.58–5.56)		(0.64–6.4)

^a^ Statistically significant difference between 2014 pre-outbreak and post-outbreak turtles

^b^ Statistically significant difference between 2015 pre-outbreak and post-outbreak turtles

^c^ Statistically significant difference between 2014 and 2015 pre-outbreak turtles

^N^ Normally distributed data

^NN^ Non-normally distributed data

### Spatial epidemiology

Statistically significant clusters of necrotizing bacterial infection cases were not identified at FGNP in 2011. However, all FV3 outbreaks had significant spatio-temporal associations including a 0.16km cluster of FV3 cases from August 2^nd^–August 16^th^ 2013 at KCCP (p = 0.0005, Fig 3), a 0.03km cluster from July 15^th^–July 25^th^ 2014 at KSP (p = 0.002, Fig 4), and a 0.16km cluster from July 8^th^–July 23^th^ 2015 at KSP (p = 0.01, Fig 4).

## Discussion

The present report details four mortality events in Illinois eastern box turtles. We found that box turtle mortality occurs commonly and has the potential for reoccurrence at a single site. Pathogens were frequently detected before, during, and after mortality events; and FV3 was the most common cause of death. One mortality event was investigated in isolation, but led to the development of health assessment projects that characterized the other three outbreaks. The important findings and limitations of each event will now be reviewed.

### 2011 –Necrotizing bacterial infection at forest glen nature preserve

In April, 2011 over 50 box turtles were found dead at a single location. Remains were completely skeletonized, precluding a definitive diagnosis. However, FV3 was not detected in banked shells, lowering the likelihood of one known cause of mass mortality [37]. Almost half of the live turtles were diagnosed with a necrotizing bacterial infection (NBI) based on histopathology (N = 2), consistent culture isolates (N = 4), and compatible clinical signs (N = 5). NBI are characterized by rapidly progressive soft tissue necrosis due to direct cellular damage from bacterial toxins, thrombosis, and the development of compartment syndrome [44–45]. Sepsis and toxemia can occur rapidly, contributing to a high mortality rate. NBI can develop secondary to relatively minor trauma such as needle sticks and insect bites, but in up to 50% of human cases no underlying cause is identified [44,46–47].

In human medicine, NBI are divided into three categories: Type I is due to polymicrobial infections and is most common, while Types II and III are due to monomicrobial infections with group A *Streptococcus* sp. or methicillin-resistant *Staphylococcus aureus* and *Clostridium* sp., *Vibrio* sp., *Aeromonas* sp., and others, respectively. In Type I infections, common aerobic isolates include *Streptococcus* sp., *Staphylococcus* spp., *Enterococcus* spp. and Enterobacteriaceae. *Bacteroides* sp. are the most common anaerobic isolates [44–47].

The syndrome identified in box turtles was clinically, histologically, and microbiologically consistent with a polymicrobial NBI. This diagnosis is rare in veterinary medicine, and even more unusual in reptiles. To the authors’ knowledge, the only report of NBI in reptiles involves a series of monomicrobial *Streptococcus agalactiae* infections in farmed saltwater crocodiles (*Crocodylus porosus*), suspected to be associated with an environmental bacterial source [48]. An environmental source of bacteria transmitted by inoculation of wounds or through the bite of insect vectors was also suspected in the present report. However, this cannot be confirmed because environmental samples were not collected for culture. Furthermore, interpretation of wound culture results should be approached with caution, as the causative agent may have either been outcompeted in culture, or may have been an organism which does not routinely grow in standard culture conditions [49]. The end of the outbreak was also poorly defined due to a lack of follow-up sampling at FGNP within the same year. While a novel disease syndrome was discovered during this mortality investigation, late recognition and lack of baseline health assessments reduced the quantity and quality of diagnostic information obtained. A routine health surveillance program was established at FGNP in 2012 and no additional mass mortalities have been identified, though an additional case of NBI was diagnosed and successfully treated in 2017.

### FV3 at kennekuk Cove County Park (2013) and kickapoo state park (2014, 2015)

Mortality events attributed to FV3 were identified at two sites in Illinois over the course of three years. FV3 has previously been associated with free-living eastern box turtle mortality events in Kentucky, Indiana, Tennessee, Pennsylvania, New York, Georgia, West Virginia, and Maryland, encompassing approximately the entire range of the species [22–24,26,50–53]. FV3 is typically diagnosed in box turtles during their active season (approximately April–October), but expected outbreak durations are poorly defined. In Kentucky, a suspected FV3 mortality event began in June and lasted for approximately 43 days [23]. In Pennsylvania, recurrent mortality events began in late July or August and lasted approximately 60 days [24]. These reports used radio-telemetry to closely monitor turtles, facilitating early recognition and response to mortality events.

The FV3 outbreak at KCCP occurred in August and lasted 30 days, while both events at KSP were identified in July and ranged from 9–20 days. Outbreak identification in the present study was opportunistic, and the true duration of FV3 mortality events may be underestimated. However, all observed FV3 cases clustered both spatially and temporally, which is consistent with previous reports [23–24]. Detection of FV3 during outbreaks, but not during pre and post outbreak surveillance, supports classification of FV3 as an epidemic disease in our study populations.

It is hypothesized that FV3 epidemics in box turtles are due to spillover from infected amphibians. This theory is supported by transmission studies documenting inter-class spread, a characteristic 3–4 week time lag between natural amphibian and chelonian infections, and field observations documenting concurrent FV3 infection in sympatric amphibians and box turtles [51,54–55]. Ranavirus-infected box turtles have been reported to congregate in wetlands, suggesting a potential route for contact and disease transmission between infected amphibians and turtles [23–24]. The present report documented significantly higher odds of FV3 detection in box turtles occupying moist microhabitats used by FV3 positive amphibians. This lends statistical credence to anecdotal observations, supports microhabitat use as a predictor of FV3 infection in box turtles, and identifies ephemeral wetlands as a possible target for disease management interventions.

While FV3 was the ultimate cause of death in the outbreaks from 2013–2015, co-pathogen infections can modulate ranavirus mortality rates in amphibians and box turtles [25,56] and may have impacted the timing of infection or susceptibility of the animals in this report. In one study, captive eastern box turtles naturally co-infected with FV3, TerHV1, and BTMyco had a higher survival rate (63%) than turtles infected with FV3 alone (50%) [25]. Due to the cross-sectional nature of the present study, mortality outcomes were unknown for most FV3 cases and the impact of co-pathogen infections on mortality rates could not be assessed directly. Anecdotally, blepharoedema and ocular discharge were identified more commonly in turtles with co-pathogen detection compared to those with single pathogens; though an improvement in sample size would be needed for rigorous statistical assessment of these findings. There were no consistent patterns of infection during any of the outbreak groupings, and neither single nor co-pathogen status were significant predictors of FV3 detection. Co-pathogen detection appears common preceding, during, and after box turtle mortality events, and the influence of co-pathogen infections on mortality outcomes warrants further study in both captive and natural settings.

Clinical signs of FV3 infection include blepharoedema, ocular, oral, and/or nasal discharge, oral plaques, respiratory distress, depression, cutaneous abscessation or erosions, cloacal plaques, edema, and death, though several of these signs are variably reported [21–22,25–26,50,52–53,57]. The only physical exam abnormalities significantly associated with FV3 detection in the present study were oral plaques and nasal discharge, but these signs were not consistently present between sites and years. Importantly, these clinical signs were indistinguishable from those associated with detection of BTMyco during the 2013 outbreak, and with previous reports of BTMyco infection in box turtles [25,29]. Clinical signs alone are not a reliable method to diagnose FV3, and confirmatory testing must be pursued when investigating upper respiratory disease outbreaks in wild box turtles.

Clinical pathology changes in FV3 positive box turtles were limited to elevations in the heterophil count and the HL ratio (driven primarily by increased heterophils). Intracytoplasmic inclusion bodies consistent with FV3 infection are sometimes visualized in box turtle blood smears, but they were not observed in affected turtles in the present report [52]. Red-eared sliders also show minimal hematologic changes during FV3 infection, with transient elevations in WBC and a persistent decrease in TS over time despite overwhelming systemic inflammation [58]. While hematology findings for FV3 positive chelonians are infrequently reported, available information indicates that complete blood counts have limited diagnostic utility for FV3 infection unless they are performed serially or at the population level to detect subtle changes [58]. It is possible that clinically useful information may be gained from evaluation of additional CBCs in FV3-positive box turtles, but an increase in sample size is needed.

Ranavirus outbreaks can recur in herptile populations over the course of multiple years, though the mechanisms for persistence are unknown [24,50,59]. In the present report, recurrent FV3 infection was observed within the same 0.2km area in KSP during 2014 and 2015. This location encompasses several ephemeral ponds used for amphibian breeding and multiple small fields heavily invaded by autumn olive (*Elaeagnus umbellata*) shrubs. These invasive plants are hypothesized to degrade box turtle habitat quality by eliminating access to fields, a favored site for foraging, thermoregulating, and nesting [20]. It is unclear whether the recurrent ranavirus outbreaks may be related to poor habitat quality, habitat fragmentation, frequent contact with ephemeral ponds containing infectious amphibians, increased exposure to human disturbance in public parks, or other factors. However, one of the most useful outcomes of mortality investigation is identifying populations for targeted study, and the 2014 and 2015 mortality investigations at KSP accomplished this. We have since launched both a box turtle ratio-telemetry study and a sympatric amphibian health surveillance program focusing on the recurrent outbreak site. These projects will facilitate the collection of baseline health data and the characterization of future natural mortality events incorporating individual, environmental, and community ecological factors.

Potential limitations in the present study include small sample size, incomplete hematology data, and reliance upon molecular diagnostics for pathogen detection. Sample size is commonly problematic in wildlife mortality studies, especially for cryptic species [10–11]. The use of trained dogs has been proposed as a means to improve carcass recovery, and while we rely upon a canine search team to locate live turtles, their recovery of deceased turtles is limited [34,60]. Increasing the number of people involved in human searches, the search frequency, or investing in carcass recovery dogs may improve the number of turtles recovered during future mortality events, though imperfect detection will likely remain a significant hurdle.

The use of molecular diagnostics for detecting wildlife diseases has rapidly expanded in recent years due to ease of use and affordability, though the use of PCR as a sole diagnostic test for amphibian ranavirus infections has recently been questioned [61]. PCR-based tests are highly sensitive, and validated TaqMan primer-probes are quite specific. All qPCR assays used in this manuscript have been validated for use in box turtles, and the risk of non-specific target amplification is considered incredibly low [38–40]. Inability to distinguish between infectious and non-infectious pathogen presence is the main limitation of a TaqMan qPCR assay, and the possibility of detecting non-infectious pathogen DNA cannot be fully excluded in the present study.

Confirmatory non-invasive diagnostic tests including virus isolation for the detection of TerHV1, FV3, and BTADV, and culture to confirm the presence of live *Mycoplasma* sp. were either not used or not consistently applied in this study. TerHV1 and BTADV have never been successfully isolated, and Mycoplasma organisms are notoriously difficult to culture [62]. An ELISA is available for *Mycoplasma agassizii*, but this test has not been validated for use in box turtles [63]. Another ELISA is available for FV3, but its sensitivity and specificity have not been objectively assessed during active infections in box turtles [64]. Other means of confirming pathogen presence (histopathology, immunohistochemistry, electron microscopy, etc.) require invasive tissue sampling, which was outside the scope of this study. Molecular diagnostics were considered adequate to achieve the study goals given the planned sampling methods, the unpredictable nature of mortality events, and the limited availability of validated methods for minimally-invasive diagnosis of box turtle infectious diseases.

The present study provides valuable descriptions of four natural mortality events in Eastern box turtles, including the first report of necrotizing bacterial infection in this species. Several predictors for FV3 detection were confirmed over the course of three outbreaks, including use of moist microhabitats, presence of oral plaques and nasal discharge, heterophil count, and the HL ratio. Single and co-pathogen detection was common before, during, and after mortality events. Finally, mortality investigation initiated longitudinal box turtle health surveillance projects at each study site. Successful mortality investigations in free-living chelonians require significant personnel time and financial investment, but the information gained is vital for planning future studies and management interventions. Continued investigation of health, disease, and death in free-living box turtles and their communities will improve baseline knowledge of morbidity and mortality, identify threats to survival, and promote the formation of effective conservation strategies.

## Supporting information

S1 File Original data used to generate this manuscript. (CSV)

## References

[1] Blehert DS, Hicks AC, Behr M, Meteyer CU, Berlowski-Zier BM, Buckles EL, et al Bat white-nose syndrome: an emerging fungal pathogen? Science. 2009 1 9;323(5911):227 doi: 10.1126/science.1163874 18974316 10.1126/science.1163874

[2] LaDeau SL, Kilpatrick AM, Marra PP. West Nile virus emergence and large-scale declines of North American bird populations. Nature. 2007 6 7;447(7145):710–3. doi: 10.1038/nature05829 17507930 10.1038/nature05829

[3] Crane M, Hyatt A. Viruses of fish: an overview of significant pathogens. Viruses. 2011 11;3(11):2025–46. doi: 10.3390/v3112025 22163333 10.3390/v3112025 PMC3230840

[4] Berger L, Speare R, Daszak P, Green DE, Cunningham AA, Goggin CL, et al Chytridiomycosis causes amphibian mortality associated with population declines in the rain forests of Australia and Central America. Proc Natl Acad Sci U S A. 1998 7 21;95(15):9031–6. 9671799 10.1073/pnas.95.15.9031 PMC21197

[5] Martel A, Sluijs AS Der, Blooi M, Bert W, Ducatelle R, Fisher MC. *Batrachochytrium salamandrivorans* sp. nov. causes lethal chytridiomycosis in amphibians. Proc Natl Acad Sci U S A. 2013 9 17;110(38):15325–9. doi: 10.1073/pnas.1307356110 24003137 10.1073/pnas.1307356110 PMC3780879

[6] Clark RW, Marchand MN, Clifford BJ, Stechert R, Stephens S. Decline of an isolated timber rattlesnake (*Crotalus horridus*) population: Interactions between climate change, disease, and loss of genetic diversity. Biol Conserv. 2011;144:886–891.

[7] Schloegel LM, Hero JM, Berger L, Speare R, McDonald K, Daszak P. The decline of the sharp-snouted day frog (*Taudactylus acutirostris*): the first documented case of extinction by infection in a free-ranging wildlife species? EcoHealth. 2006;3:35–40.

[8] Daszak P, Cunningham AA, Hyatt AD. Anthropogenic environmental change and the emergence of infectious diseases in wildlife. Acta Trop. 2001 2 23;78(2):103–16. 11230820 10.1016/s0001-706x(00)00179-0

[9] Taylor LH, Latham SM, Woolhouse MEJ. Risk factors for human disease emergence. Phil Trans R Soc Lond B Biol Sci. 2001 7 29;356(1411):983–9. 11516376 10.1098/rstb.2001.0888 PMC1088493

[10] Wobeser GA. Essentials of disease in wild animals Ames: Blackwell Publishing; 2006.

[11] Sleeman JM, Brand CJ, Wright SD. Strategies for wildlife disease surveillance In: Aguirre AA, Ostfeld RS, Daszak P, editors. New directions in conservation medicine applied cases of ecological health. New York: Oxford University Press Inc; 2012 pp. 539–51.

[12] Ryser-Degiorgis M-P. Wildlife health investigations: needs, challenges and recommendations. BMC Vet Res. 2013 11 4;9(223). doi: 10.1186/1746-6148-9-223 24188616 10.1186/1746-6148-9-223 PMC4228302

[13] Mörner T, Obendorf DL, Artois M, Woodford MH. Surveillance and monitoring of wildlife diseases. Rev Sci Tech. 2002;21(1):67–76. 11974631 10.20506/rst.21.1.1321

[14] Stallknecht DE. Impediments to wildlife disease surveillance, research, and diagnostics. Curr Top Microbiol Immunol. 2007;315:445–61. 17848074 10.1007/978-3-540-70962-6_17

[15] Artois M, Bengis R, Delahay RJ, Duchêne M, Duff JP, Ferroglio E, et al Wildlife disease surveillance and monitoring In: Delahay RJ, Smith GC, Hutchings MR, editors. Management of disease in wild mammals. Tokyo: Springer Japan; 2009 pp. 187–213.

[16] Brand CJ. Wildlife mortality investigation and disease research: contributions of the USGS national wildlife health center to endangered species management and recovery. Ecohealth. 2013;10:446–54. doi: 10.1007/s10393-013-0897-4 24419670 10.1007/s10393-013-0897-4 PMC3938848

[17] Turtle Taxonomy Working Group [Rhodin AGJ, Iverson JB, Bour R, Fritz U, Georges A, Shaffer HB, et al]. Turtles of the world: annotated checklist and atlas of taxonomy, synonymy, distribution, and conservation status (8th ed.). Chelonian Research Monographs. 2017;7:1–292. doi: 10.3854/crm.7.checklist.atlas.v8.2017

[18] Heppell SS. Application of life-history theory and population model analysis to turtle conservation. Copeia. 1998;2:367–75.

[19] Stacy BA, Wolf DA, Wellehan JFX. Large-scale predation by river otters (*Lontra canadensis*) on Florida cooter (*Pseudemys floridana*) and Florida softshell turtles (*Apalone ferox*). J Wildl Dis. 2014 10;50(4):906–10. doi: 10.7589/2013-10-271 25098299 10.7589/2013-10-271

[20] Dodd CK. North American box turtles: a natural history Norman: Univ. of Oklahoma Press; 2001.

[21] De Voe R, Geissler K, Elmore S, Rotstein D, Lewbart G, Guy J. Ranavirus-associated morbidity and mortality in a group of captive eastern box turtles (*Terrapene carolina carolina*). J Zoo Wildl Med. 2004 12;35(4):534–43. doi: 10.1638/03-037 15732597 10.1638/03-037

[22] Johnson AJ, Pessier AP, Wellehan JF, Childress A, Norton TM, Stedman NL, et al Ranavirus infection of free-ranging and captive box turtles and tortoises in the United States. J Wildl Dis. 2008 10;44(4):851–63. doi: 10.7589/0090-3558-44.4.851 18957641 10.7589/0090-3558-44.4.851

[23] Agha M, Price SJ, Nowakowski AJ, Augustine B, Todd BD. Mass mortality of eastern box turtles with upper respiratory disease following atypical cold weather. Dis Aquat Org. 2017 4 20;124(2):91–100. doi: 10.3354/dao03122 28425422 10.3354/dao03122

[24] Belzer WR, Seibert S. A natural history of ranavirus in an eastern box turtle population. Turtle and Tortoise Newsletter. 2011;15:18–25.

[25] Sim RR, Allender MC, Crawford LK, Wack AN, Murphy KJ, Mankowski JL, et al Ranavirus epizootic in captive eastern box turtles (*Terrapene carolina carolina*) with concurrent herpesvirus and mycoplasma infection: management and monitoring. J Zoo Wildl Med. 2016 3;47(1):256–70. doi: 10.1638/2015-0048.1 27010285 10.1638/2015-0048.1

[26] Farnsworth SD, Seigel RA. Responses, movements, and survival of relocated box turtles during construction of the intercounty connector highway in Maryland. Transp Res Rec. 2013;2362:1–8.

[27] Kimble SJ, Karna AK, Johnson AJ, Hoverman JT, Williams RN. Mosquitoes as a potential vector of ranavirus transmission in terrestrial turtles. *EcoHealth*. 2015 6;12(2):334–8. doi: 10.1007/s10393-014-0974-3 25212726 10.1007/s10393-014-0974-3

[28] Sim RR, Norton TM, Bronson E, Allender MC, Stedman N, Childress AL, et al Identification of a novel herpesvirus in captive eastern box turtles (*Terrapene carolina carolina*). Vet Microbiol. 2015 2 25;175(2–4):218–23. doi: 10.1016/j.vetmic.2014.11.029 25575878 10.1016/j.vetmic.2014.11.029

[29] Feldman SH, Wimsatt J, Marchang RE, Johnson AJ, Brown W, Mitchell JC, et al A novel mycoplasma detected in association with upper respiratory disease syndrome in free-ranging eastern box turtles (*Terrapene carolina carolina*) in Virginia. J Wildl Dis. 2006 4;42(2):279–89. doi: 10.7589/0090-3558-42.2.279 16870850 10.7589/0090-3558-42.2.279

[30] Ossiboff RJ, Raphael BL, Ammazzalorso AD, Seimon TA, Niederriter H, Zarate B, et al A mycoplasma species of Emydidae turtles in the northeastern USA. J Wildl Dis. 2015 4;51(2):466–70. doi: 10.7589/2014-04-086 25574806 10.7589/2014-04-086

[31] Doszpoly A, Wellehan JFX, Childress AL, Tarján ZL, Kovács ER. Partial characterization of a new adenovirus lineage discovered in testudinoid turtles. Infect Genet Evol. 2013 7;17:106–12. doi: 10.1016/j.meegid.2013.03.049 23567817 10.1016/j.meegid.2013.03.049

[32] Farkas SL, Gal J. Adenovirus and mycoplasma infection in an ornate box turtle (*Terrapene ornata ornata*) in Hungary. Vet Microbiol. 2009 7 2;138(1–2):169–73. doi: 10.1016/j.vetmic.2009.03.016 19375875 10.1016/j.vetmic.2009.03.016

[33] Kane LP, Allender MC, Archer G, Dzhaman E, Pauley J, Moore AR, et al Prevalence of Terrapene herpesvirus 1 in free-ranging eastern box turtles (*Terrapene carolina carolina*) in Tennesee and Illinois, USA. J Wildl Dis. 2017 4;53(2):285–95. doi: 10.7589/2016-06-138 28099078 10.7589/2016-06-138

[34] Boers K, Leister K, Byrd J, Band M, Phillips CA, Allender MC. Capture effort, rate, demographics, and potential for disease transmission in wild eastern box turtles (*Terrapene carolina carolina*) captured through canine directed searches. Herpetol Rev. 2017;48(2):300–4.

[35] Cagle FR. A system of marking turtles for future identification. Copeia. 1939;3:170–173.

[36] Jousimies-Somer H, Summanen P, Citron DM, Baron EJ, Wexler HM, Finegold SM, editors. Wadsworth-KTL Anaerobic Bacteriology Manual. 6th ed. Belmont (CA): Star Publishing Company; 2003.

[37] Butkus CE, Allender MC, Phillips CA, Adamovicz LA. Diagnosis of ranavirus using bone marrow harvested from mortality events in Eastern box turtles (*Terrapene carolina carolina*). J Zoo Wildl Med. 2017;48(4): 1210–1214. doi: 10.1638/2017-0098.1 29297832 10.1638/2017-0098.1

[38] Allender MC, Bunick D, Mitchell MA. Development and validation of TaqMan quantitative PCR for detection of frog virus 3-like virus in eastern box turtles (*Terrapene carolina carolina*). J Virol Methods. 2013 3;188(1–2):121–5. doi: 10.1016/j.jviromet.2012.12.012 23274753 10.1016/j.jviromet.2012.12.012

[39] Kane LP, Bunick D, Abd-Eldaim M, Dzhaman E, Allender MC. Development and validation of quantitative PCR for detection of Terrapene herpesvirus 1 utilizing free-ranging eastern box turtles(*Terrapene carolina carolina*). J Virol Methods. 2016 6;232:57–61. doi: 10.1016/j.jviromet.2016.02.002 26874287 10.1016/j.jviromet.2016.02.002

[40] Blum SA, Norton TM, Deem SL, Hall N, Fleming G, Wellehan JFX. Development of a quantitative PCR assay for a novel adenovirus in the box turtle (Terrapene carolina). Proceedings of the Association of Reptilian and Amphibian Veterinarians; 2014 Oct 18–14; Orlando, FL. p. 17.

[41] Hyatt AD, Gould AR, Zupanovic Z, Cunningham AA, Hengstberger S, Whittington RJ, Kattenbelt J, Coupar BE. Comparative studies of piscine and amphibian iridoviruses. Arch Virol. 2000;145(2):301–31. 10752555 10.1007/s007050050025

[42] Kulldorff M. A spatial scan statistic. Commun Stat Theory Methods. 1997;26:1481–96.

[43] R Core Team. R: A language and environment for statistical computing R Foundation for Statistical Computing, Vienna, Austria 2013.

[44] Sarani B, Strong M, Pascual J, Schwab CW. Necrotizing fasciitis: current concepts and review of the literature. J Am Coll Surg. 2009 2;208(2):279–88. doi: 10.1016/j.jamcollsurg.2008.10.032 19228540 10.1016/j.jamcollsurg.2008.10.032

[45] Sartelli M, Malangoni MA, May AK, Viale P, Kao LS, Catena F, Ansaloni L, et al World Society of Emergency Surgery (WSES) guidelines for management of skin and soft tissue infections. World J Emerg Surg. 2014 11 18;9(1):1–57. 25422671 10.1186/1749-7922-9-57 PMC4242587

[46] Edlich RF, Cross CL, Dahlstrom JJ, Long WB. Modern concepts of the diagnosis and treatment of necrotizing fasciitis. J Emerg Med. 2010 8;39(2):261–5. doi: 10.1016/j.jemermed.2008.06.024 19081698 10.1016/j.jemermed.2008.06.024

[47] Phan HH, Cocanour CS. Necrotizing soft tissue infections in the intensive care unit. Crit Care Med. 2010;38(9):S460–S468. 20724879 10.1097/CCM.0b013e3181ec667f

[48] Bishop EJ, Shilton C, Benedict S, Kong F, Gilbert GL, Gal D, et al Necrotizing fasciitis in captive juvenile *Crocodylus porosus* caused by *Streptococcus agalactiae*: an outbreak and review of the animal and human literature. Epidemiol Infect. 2007 11;135(8):1248–55. doi: 10.1017/S0950268807008515 17445318 10.1017/S0950268807008515 PMC2870709

[49] Rappe MS, Giovannoni SJ. The uncultured microbial majority. Annu Rev Microbiol. 2003 57:369–94. doi: 10.1146/annurev.micro.57.030502.090759 14527284 10.1146/annurev.micro.57.030502.090759

[50] Kimble SJA, Johnson AJ, Williams RN, Hoverman JT. A severe ranavirus outbreak in captive, wild-caught box turtles. EcoHealth. 2017;https://doi.org/10.1007/s10393-017-1263-8. 10.1007/s10393-017-1263-8 28766064

[51] Currylow AF, Johnson AJ, Williams RN. Evidence of ranavirus infections among sympatric larval amphibians and box turtles. J Herpetol. 2014;48:117–121.

[52] Allender MC, Fry MM, Irizarry AR, Craig L, Johnson AJ, Jones M. Intracytoplasmic inclusions in circulating leukocytes from an eastern box turtle (*Terrapene carolina carolina*) with iridoviral infection. J Wildl Dis. 2006 7;42(3):677–684. doi: 10.7589/0090-3558-42.3.677 17092902 10.7589/0090-3558-42.3.677

[53] Ruder MG, Allison AB, Miller DL, Keel MK. Pathology in practice. Ranavirus infection. J Am Vet Med Assoc. 2010;237(7):783–785. doi: 10.2460/javma.237.7.783 20919842 10.2460/javma.237.7.783

[54] Brenes R, Gray MJ, Waltzek TB, Wilkes RP, Miller DL. Transmission of ranavirus between ectothermic vertebrate hosts. PLoS One. 2014 3 25;9(3):e92476 doi: 10.1371/journal.pone.0092476 24667325 10.1371/journal.pone.0092476 PMC3965414

[55] Brunner JL, Storfer A, Gray MJ, Hoverman JT. Ranavirus ecology and evolution: from epidemiology to extinction In: Gray MJ, Chinchar VG editors. Ranaviruses lethal pathogens of ectothermic vertebrates. Heidelberg: Springer; 2015 pp. 71–104.

[56] Wuerthner VP, Hua J, Hoverman JT. The benefits of coinfection: trematodes alter disease outcomes associated with virus infection. J Anim Ecol. 2017 7;86(4):921–31. doi: 10.1111/1365-2656.12665 28317105 10.1111/1365-2656.12665

[57] Johnson AJ, Pessier AP, Jacobson ER. Experimental transmission and induction of ranaviral disease in western ornate box turtles (*Terrapene ornata ornata*) and red-eared sliders (*Trachemys scripta elegans*). Vet Pathol. 2007 5;44(3):285–97. doi: 10.1354/vp.44-3-285 17491069 10.1354/vp.44-3-285

[58] Allender MC, Mitchell MA. Hematologic response to experimental infections of frog virus 3-like virus in red-eared sliders (*Trachemys scripta elegans*). J Herp Med Surg. 2013;23(1–2):25–31.

[59] Teacher AGF, Cunningham AA, Garner TWJ. Assessing the long term impact of ranavirus infection in wild common frog populations. Anim Conserv. 2010;13:514–22.

[60] Homan HJ, Linz G, Peer BD. Dogs increase recovery of passerine carcasses in dense vegetation. Wildl Soc Bull. 2001;29(1):292–6.

[61] Black Y, Meredith A, Price SJ. Detection and reporting of ranavirus in amphibians: evaluation of the roles of the World Organization for Animal Health and the published literature. J Wildl Dis. 2017 7;53(3):509–20. doi: 10.7589/2016-08-176 28402726 10.7589/2016-08-176

[62] Brown MB, Schumacher IM, Klein PA, Harris K, Correll T, Jacobson ER. *Mycoplasma agassizii* causes upper respiratory tract disease in the desert tortoise. Infect Immun. 1994;62(10):4580–6. 7927724 10.1128/iai.62.10.4580-4586.1994 PMC303146

[63] Schumacher IM, Brown MB, Jacobson ER, Collins BR, Klein PA. Detection of antibodies to a pathogenic mycoplasma in desert tortoises (*Gopherus agassizii*) with upper respiratory tract disease. J Clin Microbiol. 1993;31:1454–60. 8314986 10.1128/jcm.31.6.1454-1460.1993 PMC265561

[64] Johnson AJ, Wendland L, Norton TM, Belzer B, Jacobson ER. Development and use of an indirect enzyme-linked immunosorbent assay for detection of iridovirus exposure in gopher tortoises (*Gopherus polyphemus*) and eastern box turtles (*Terrapene carolina carolina*). Vet Microbiol. 2010;142(3–4):160–7. doi: 10.1016/j.vetmic.2009.09.059 19931321 10.1016/j.vetmic.2009.09.059

